# Revision of visual impairment definitions in the International Statistical Classification of Diseases

**DOI:** 10.1186/1741-7015-4-7

**Published:** 2006-03-16

**Authors:** Lalit Dandona, Rakhi Dandona

**Affiliations:** 1 Health Studies Area, Centre for Human Development, Administrative Staff College of India, Hyderabad, India

## Abstract

**Background:**

The existing definitions of visual impairment in the International Statistical Classification of Diseases are based on recommendations made over 30 years ago. New data and knowledge related to visual impairment that have accumulated over this period suggest that these definitions need to be revised.

**Discussion:**

Three major issues need to be addressed in the revision of these definitions. First, the existing definitions are based on best-corrected visual acuity, which exclude uncorrected refractive error as a cause of visual impairment, leading to substantial underestimation of the total visual impairment burden by about 38%. Second, the cut-off level of visual impairment to define blindness in the International Statistical Classification of Diseases is visual acuity less than 3/60 in the better eye, but with increasing human development the visual acuity requirements are also increasing, suggesting that a level less than 6/60 be used to define blindness. Third, the International Statistical Classification of Diseases uses the term 'low vision' for visual impairment level less than blindness, which causes confusion with the common use of this term for uncorrectable vision requiring aids or rehabilitation, suggesting that alternative terms such as moderate and mild visual impairment would be more appropriate for visual impairment less severe than blindness. We propose a revision of the definitions of visual impairment in the International Statistical Classification of Diseases that addresses these three issues. According to these revised definitions, the number of blind persons in the world defined as presenting visual acuity less than 6/60 in the better eye would be about 57 million as compared with the World Health Organization estimate of 37 million using the existing International Statistical Classification of Diseases definition of best-corrected visual acuity less than 3/60 in the better eye, and the number of persons in the world with moderate visual impairment defined as presenting visual acuity less than 6/18 to 6/60 in the better eye would be about 202 million as compared with the World Health Organization estimate of 124 million persons with low vision defined as best-corrected visual acuity less than 6/18 to 3/60 in the better eye.

**Conclusion:**

Our suggested revision of the visual impairment definitions in the International Statistical Classification of Diseases takes into account advances in the understanding of visual impairment. This revised classification seems more appropriate for estimating and tracking visual impairment in the countries and regions of the world than the existing classification in the International Statistical Classification of Diseases.

## Background

The currently available version of the tenth revision of the International Statistical Classification of Diseases and Related Health Problems (ICD) defines visual impairment categories primarily on the basis of recommendations made by a World Health Organization (WHO) Study Group in 1972 [1]. Since these recommendations of over three decades ago, there have been substantial studies on the distribution of blindness and less severe visual impairment in populations worldwide. These studies have incrementally suggested a more nuanced understanding of visual impairment and of how it should be defined to comprehend its actual burden, as revealed by recent reviews [2-6]. As ICD is considered the standard worldwide classification, the ICD definitions of visual impairment are used most often for worldwide estimates of visual impairment [4,5]. However, several issues with these ICD definitions need to be addressed for better clarity and utilisation, including some that have been referred to previously in the literature [2-5,7-10]. In this paper, we bring together the major issues regarding the ICD definitions of visual impairment that would benefit from revision. On the basis of current understanding of visual impairment, we propose modifications in the ICD definitions that might enable their better practical utilisation for classification and estimation of the different levels of visual impairment worldwide.

## Discussion

The current categories of visual impairment in ICD are shown in Table 1, and their use to classify different levels of visual impairment is shown in Table 2. We identified three major issues in this ICD classification that need to be addressed: use of best-corrected or presenting visual acuity, cut-off level to define blindness, and appropriateness of the term 'low vision'.

### Best-corrected or presenting visual acuity

There is increasing consensus that the use of best-corrected visual acuity to assess the burden of visual impairment in a population is inappropriate as it misses visual impairment caused by uncorrected refractive error [2,3,5-7]. The use of presenting visual acuity, that is acuity with whatever refractive correction the person is using, is more appropriate as it enables uncorrected refractive error to be included as a cause of visual impairment. Our review of the published data suggests that there may be about 98 million persons with visual impairment due to uncorrected refractive error worldwide [6], in addition to the 161 million persons estimated by WHO to have visual impairment with best-corrected visual acuity [5]. This implies that of the estimated total 259 million persons worldwide with visual impairment, 38% would be erroneously excluded with the best-corrected acuity definition of visual impairment. This is particularly ironic as uncorrected refractive error is the most easily treatable cause of visual impairment, usually with a simple pair of spectacles. Perhaps because it is so easily treatable, it was not initially considered worthy of qualifying as a "cause" of visual impairment in the ICD definition based on recommendations made over 30 years ago [1]. However, the assumption in the ICD definition – that persons with poor vision due to uncorrected refractive error are not visually impaired because they could have better vision if they had simple refractive correction with spectacles – seems misplaced, as they have poor vision as long as they do not get refractive correction. If extended to cataract, this anomalous assumption could imply that because most persons visually impaired due to cataract could potentially have their vision restored with cataract surgery, they are not visually impaired because best correction (in this case cataract surgery) would probably restore their vision. A recent estimate suggests that uncorrected refractive error is the most common cause of visual impairment in the world [6], emphasising the urgent need to replace best-corrected visual acuity with presenting visual acuity for defining visual impairment in the ICD classification. This will avoid the huge underestimation of the actual visual impairment burden that occurs with the existing ICD definition.

For perspective, it is interesting to note that a historical analysis has suggested that the invention of eye glasses to improve vision was one of the few most important contributors to human development over the past several centuries [11]. This further underscores the need not to overlook uncorrected refractive error in the definition of visual impairment.

Since definitions of visual impairment are based on distance visual acuity, it is important to note certain features of visual impairment related to uncorrected refractive error. First, a portion of the persons who qualify as visually impaired due to uncorrected refractive error would have good near vision. Such impairment may be less disabling than the visual impairment that causes poor vision at both distance and near. More needs to be understood about the extent to which the disabling effects of these two types of visual impairment differ. Second, blindness due to uncorrected natural refractive error sets in at a young age, resulting in many more blind years suffered per person than with most other major causes of blindness that usually set in at a later age [12]. Third, disability also occurs due to uncorrected refractive error related to aging that causes difficulty in seeing at near, which usually sets in around the age of 40 years and is referred to as presbyopia. But adequate data are not available yet to suggest how this could be included in the visual impairment definitions. This deficiency would need to be addressed in the future. The first of these three features of visual impairment due to uncorrected refractive error would suggest a relatively lower disability, whereas the latter two would suggest higher unaccounted disability. Clearly, it would be useful to generate further knowledge indicating how these features could be taken into account while assessing visual impairment due to uncorrected refractive error.

### Visual acuity level to define blindness

Because the ICD definition and WHO recommend use of a visual acuity level less than 3/60 to define blindness [1], many population-based surveys from less developed countries have reported blindness rates with this definition in the past [4]. There are exceptions such as India, where a visual acuity level less than 6/60 is used to define blindness [12], and recent reports from other less developed countries covering African and Chinese populations that have used visual acuity less than 6/60 to define blindness [13-16]. The more developed countries have often used visual acuity level less than 6/60 to define blindness [17,18]; the United States uses visual acuity less than or equal to 6/60 for this definition [19]. The level of human development in less developed countries has been increasing over the past few decades, as indicated by increases in life expectancy, literacy and income [20]. Since the recommendation to use visual acuity level less than 3/60 to define blindness some three decades ago [1], higher levels of vision are now required for optimal functioning even in less developed countries because of the increasing complexity of daily tasks, prompting suggestions for using a less severe level of visual impairment to define blindness [8]. We therefore suggest that a uniform definition of blindness for both the less and more developed countries as presenting visual acuity less than 6/60 is now more appropriate than the 3/60 acuity level used by the ICD definition (Table 3). In the existing ICD visual impairment categories, there is no visual field loss corresponding to visual acuity less than 6/60 to 3/60 (Table 1) [1]. It has previously been suggested that for visual acuity level of 6/60 the equivalent central visual field of 20° seems appropriate [21,22], and we propose this for inclusion in the ICD classification (Table 3).

Additionally, visual acuity less than 6/12 is often used in more developed countries to define visual impairment, as this level of vision is considered necessary for daily tasks [18,19]. Using logic similar to that used above for blindness, the increasing complexity of daily tasks even in less developed countries would require better vision with the passage of time. We therefore suggest that it would be useful to have a category of mild visual impairment in the ICD classification for presenting visual acuity less than 6/12 to 6/18 (Table 3).

### The term 'low vision'

A WHO consultation has suggested "a person with low vision as one who has impairment of visual functioning even after treatment and/or refractive correction, and has a visual acuity of less than 6/18 to light perception, or a visual field of less than 10° from the point of fixation, but who uses, or is potentially able to use, vision for the planning and/or execution of a task" [9]. This definition of 'low vision' identifies persons who have poor vision after therapy and would potentially benefit from special low vision aids or rehabilitation to enhance their quality of life [10]. This seems a more appropriate use of the term 'low vision', which is evident from the common use of the term 'Low vision clinics' around the world for clinics that provide aids and rehabilitative services to such patients. In the ICD classification, the term 'low vision' is used for visual acuity less than 6/18 to 3/60 after refractive correction, which includes treatable causes such as cataract and others [1,5]. This causes confusion with the more apt use of the term 'low vision' for persons with untreatable visual impairment of a certain level who would benefit from low vision aids or rehabilitation. We therefore suggest that the term 'moderate visual impairment' be used in the ICD classification for presenting visual acuity less than 6/18 to 6/60 instead of 'low vision' for best-corrected visual acuity less than 6/18 to 3/60 (Table 3). The prefix "moderate" in this term denotes visual impairment less severe than blindness, and allows use of the term 'mild visual impairment' for presenting visual acuity less than 6/12 to 6/18.

### Implications of the suggested ICD revision

We suggest revision of the ICD classification for visual impairment to reflect the modifications in the definitions suggested above and to indicate combinations of visual impairment in the two eyes of a person that are most commonly used in practical assessments of visual impairment (Table 4). The existing ICD classification includes two combinations of binocular visual impairment that are rarely if ever used (Table 2). First, blindness in one eye and low vision in the other eye (ICD code H54.1) is not needed, as this level and low vision in both eyes (ICD code H54.2) denote the same visual impairment level in the better eye of the person, and are not used separately in practical assessments of visual impairment. Second, unspecified visual loss (H54.7) is also not needed, as unqualified visual loss in both eyes (H54.3) and unqualified visual loss in one eye (H54.6) are already covered.

Although the revision we suggest for the ICD definitions seems more consistent with current understanding of visual impairment, it is important to recognise how the new estimates of visual impairment with these revised estimates could be compared with past estimates that have used the existing definitions. For this, the following issues would have to be taken into account:

1. Defining visual impairment as presenting visual acuity less than 6/18 in the better eye would increase the number of visually impaired persons in the world to about 259 million, compared with the WHO estimate of 161 million based on the best-corrected acuity definition, an increase of 61% [6]. This increase, indicating the true burden of visual impairment less than 6/18, would vary in magnitude in different regions of the world [6].

2. Changing the definition of blindness to presenting visual acuity less than 6/60 from best-corrected visual acuity 3/60 would increase the number of blind persons. We have estimated that the number of persons worldwide with presenting visual acuity less than 3/60 in the better eye would be about 42 million, which is 14% more than the 37 million estimated by WHO with best-corrected visual acuity less than 3/60 in the better eye [6]. Data from a few recent population-based studies that reported presenting visual acuity for both the less than 6/60 and less than 3/60 levels, and which enabled these two levels to be compared clearly, suggest that there could be an increase of 34–37% for less developed countries and even higher for more developed countries, in the number of persons with presenting visual acuity less than 6/60 as compared with presenting visual acuity less than 3/60 [12,23,24]. Based on this, the number of blind persons in the world as defined by presenting visual acuity less than 6/60 in the better eye can be roughly estimated at about 57 million (Table 5).

3. The proposed moderate visual impairment, defined as presenting visual acuity less than 6/18 to 6/60, would have estimates different from the existing low vision, defined in the ICD classification as best-corrected visual acuity less than 6/18 to 3/60. We have estimated an increase of about 75% worldwide for visual acuity level of less than 6/18 to 3/60 if presenting visual acuity were used instead of best-corrected acuity [6]. On the other hand, there would be a decrease due to exclusion of the less than 6/60 to 3/60 slab from the less than 6/18 to 3/60 range, estimated to be about 6% for less developed countries and larger for more developed countries based on the limited data available from the few studies using presenting visual acuity and allowing this distinction [12,23-25]. Overall, these two opposing effects would result in a rough estimate of about 202 million persons in the world with moderate visual impairment defined as presenting visual acuity less than 6/18 to 6/60 in the better eye, compared with the WHO estimate of 124 million persons with low vision defined as best-corrected acuity less than 6/18 to 3/60 in the better eye (Table 5).

4. Very few data are available for the proposed category of mild visual impairment defined as presenting visual acuity less than 6/12 to 6/18. In a national sample of adults aged 30 years or older in Bangladesh, the prevalence of mild visual impairment was 6.46%, which would add 65% to the 9.97% prevalence of all other levels of visual impairment (presenting visual acuity less than 6/18) [23]. In a sample of adults aged 40 years or older in Victoria, Australia, the prevalence of mild visual impairment was 2.51%, which would add 146% to the 1.72% prevalence of all other levels of visual impairment [24]. In these studies, mild visual impairment in a large proportion of the persons could be improved with refractive correction.

The calculations presented above should be considered as only indicative, as they are based on limited available data. However, keeping the above issues in mind would enable informed comparisons to be made between past estimates of visual impairment and the new estimates using the proposed revised ICD classification, for assessing the changes in trends of visual impairment in countries and regions of the world.

The ICD classification has provisions for an updating and revision process when the need arises [26]. It would seem to be useful for the ICD Updating and Revision Committee to consider the update/revision suggested in this manuscript, which is based on current understanding of visual impairment, as the existing classification is based on recommendations made over three decades ago.

## Summary

• New understanding of visual impairment has become available since the recommendations made some three decades ago, on which the existing classification of visual impairment in the International Statistical Classification of Diseases is based, suggesting that this classification must be revised.

• We propose that the visual impairment definitions in the International Statistical Classification of Diseases be based on presenting visual acuity instead of best-corrected visual acuity, the visual acuity cut-off level for blindness be changed from less than 3/60 to less than 6/60, the low vision category be modified to moderate visual impairment defined as presenting visual acuity less than 6/18 to 6/60, and the category of mild visual impairment be added defined as presenting visual acuity less than 6/12 to 6/18.

• According to these revised definitions, the number of blind persons in the world defined as presenting visual acuity less than 6/60 in the better eye would be about 57 million as compared with the estimate of 37 million using the current International Statistical Classification of Diseases definition of best-corrected visual acuity less than 3/60 in the better eye, and the number of persons in the world with moderate visual impairment defined as presenting visual acuity less than 6/18 to 6/60 in the better eye would be about 202 million as compared with the estimate of 124 million persons with low vision defined as best-corrected visual acuity less than 6/18 to 3/60 in the better eye.

• The Updating and Revision Committee of the International Statistical Classification of Diseases could consider the update/revision of the classification of visual impairment suggested in this manuscript, as this seems more appropriate than the existing classification for estimating and tracking visual impairment in the countries and regions of the world.

## Competing interests

The authors declare that they have no competing interests.

## Authors' contributions

LD conceived this report, reviewed the literature and wrote the initial draft of the manuscript. RD contributed to the ideas presented and the writing of this manuscript. Both authors approved the final version of the manuscript.

## Pre-publication history

The pre-publication history for this paper can be accessed here:



## Figures and Tables

**Table 1:** Categories of severity of visual impairment according to the International Statistical Classification of Diseases [1].

**Category of visual impairment**	**Visual acuity with best possible correction**		Or central visual field*	**Classified as**
			
	Maximum less than	Minimum equal to or better than		
1	6/18 3/10 (0.3) 20/70	6/60 1/10 (0.1) 20/200		Low vision
2	6/60 1/10 (0.1) 20/200	3/60 1/20 (0.05) 20/400		Low vision
3	3/60 1/20 (0.05) 20/400	1/60 (finger counting at 1 metre) 1/50 (0.02) 5/300 (20/1200)	10° or less but more than 5°	Blindness
4	1/60 (finger counting at 1 metre) 1/50 (0.02) 5/300	Light perception	5° or less	Blindness
5	No light perception			Blindness
9	Undetermined or unspecified			Unspecified

*Visual field restriction criteria applicable even if visual acuity is better than for that category of visual impairment.

**Table 2:** Classification of visual impairment in a person according to the International Statistical Classification of Diseases [1].

**H54**	Blindness and low vision
**ICD code**	**Level of visual impairment in a person**
**H54.0**	**Blindness, both eyes** Visual impairment categories 3, 4, 5 in both eyes.
**H54.1**	**Blindness, one eye, low vision other eye** Visual impairment categories 3, 4, 5 in one eye, with categories 1 or 2 in the other eye.
**H54.2**	**Low vision, both eyes** Visual impairment categories 1 or 2 in both eyes.
**H54.3**	**Unqualified visual loss, both eyes** Visual impairment category 9 in both eyes.
**H54.4**	**Blindness, one eye** Visual impairment categories 3, 4, 5 in one eye [normal vision in other eye].
**H54.5**	**Low vision, one eye** Visual impairment categories 1 or 2 in one eye [normal vision in other eye].
**H54.6**	**Unqualified visual loss, one eye** Visual impairment category 9 in one eye [normal vision in other eye].
**H54.7**	**Unspecified visual loss** Visual impairment category 9 NOS.

**Table 3:** Our recommendation for the categories of severity of visual impairment in the International Statistical Classification of Diseases.

**Category of visual impairment**	**Presenting visual acuity**		Or central visual field*	**Classified as**
			
	Maximum less than	Minimum equal to or better than		
1	6/12 0.50 20/40	6/18 0.33 20/60		Mild visual impairment
2	6/18 0.33 20/60	6/60 0.10 20/200		Moderate visual impairment
3	6/60 0.10 20/200	3/60 0.05 20/400	20° or less but more than 10°	Blindness
4	3/60 0.05 20/400	1/60 (finger counting at 1 metre) 0.02 5/300 (20/1200)	10° or less but more than 5°	Severe blindness
5	1/60 (finger counting at 1 metre) 0.02 5/300	Light perception	5° or less	Very severe blindness
6	No light perception			Total blindness
9	Unspecified			Unspecified

*Visual field restriction criteria applicable even if visual acuity is better than for that category of visual impairment.

**Table 4:** Our recommendation for classification of visual impairment in a person in the International Statistical Classification of Diseases.

**H54**	Blindness, moderate visual impairment and mild visual impairment
**ICD code**	**Level of visual impairment in a person**
**H54.0**	**Blindness in a person** Visual impairment category 3, 4, 5 or 6 in the better eye. Following are subsets of blindness in a person: • Severe blindness – visual impairment category 4 in the better eye. • Very severe blindness – visual impairment category 5 in the better eye. • Total blindness – visual impairment category 6 in both eyes.
**H54.1**	**Moderate visual impairment in a person** Visual impairment category 2 in the better eye.
**H54.2**	**Mild visual impairment in a person** Visual impairment category 1 in the better eye.
**H54.3**	**Unspecified visual impairment in a person** Visual impairment category 9 in both eyes.
**H54.4**	**Blindness in one eye of a person** Visual impairment category 3, 4, 5 or 6 in one eye and no visual impairment in the other eye. Following are subsets of blindness in one eye of a person: • Severe blindness – visual impairment category 4 in one eye and no visual impairment in the other eye. • Very severe blindness – visual impairment category 5 in one eye and no visual impairment in the other eye. • Total blindness – visual impairment category 6 in one eye and no visual impairment in the other eye.
**H54.5**	**Moderate visual impairment in one eye of a person** Visual impairment category 2 in one eye and no visual impairment in the other eye.
**H54.6**	**Mild visual impairment in one eye of a person** Visual impairment category 1 in one eye and no visual impairment in the other eye.
**H54.7**	**Unspecified visual impairment in one eye of a person** Visual impairment category 9 in one eye and no visual impairment in the other eye.

**Table 5:** Comparison between visual impairment estimates in the world using existing definitions and estimates using revised definitions.

	**Number of persons in the world with blindness**	**Number of persons in the world with visual impairment less severe than blindness**
**Estimates made by WHO using existing ICD definitions **[5]	37 million with blindness (best-corrected visual acuity less than 3/60 in the better eye)	124 million with low vision (best-corrected visual acuity less than 6/18 to 3/60 in the better eye)
**Estimates by us using the proposed revised ICD definitions**	57 million with blindness (presenting visual acuity less than 6/60 in the better eye)	202 million with moderate visual impairment (presenting visual acuity less than 6/18 to 6/60 in the better eye)
